# SUPERFICIAL – Surface mapping of proteins via structure-based peptide library design

**DOI:** 10.1186/1471-2105-6-223

**Published:** 2005-09-09

**Authors:** Andrean Goede, Ines S Jaeger, Robert Preissner

**Affiliations:** 1 Berlin Center for Genome Based Bioinformatics, 3D Data Mining Group, Institute of Biochemistry, Charité, Monbijoustr.2, 10117 Berlin, Germany

## Abstract

**Background:**

The determination of protein surfaces and the detection of binding sites are essential to our understanding of protein-protein interactions. Such binding sites can be characterised as linear and non-linear, the non-linear sites being prevailant. Conventional mapping techniques with arrays of synthetic peptides have limitations with regard to the location of discontinuous or non-linear binding sites of proteins.

**Results:**

We present a structure-based approach to the design of peptide libraries that mimic the whole surface or a particular region of a protein. Neighbouring sequence segments are linked by short spacers to conserve local conformation. To this end, we have developed SUPERFICIAL, a program that uses protein structures as input and generates library proposals consisting of linear and non-linear peptides. This process can be influenced by a graphical user interface at different stages, from the surface computation up to the definition of spatial regions.

**Conclusion:**

Based on 3D structures, SUPERFICIAL may help to negotiate some of the existing limitations, since binding sites consisting of several linear pieces can now be detected.

## Background

In order to perform their functions, protein surfaces usually have to interact with each other. However, only accessible parts of a protein can act as binding sites [1]. Since proteins consist of polypeptide chains that fold into complex three-dimensional patterns, binding sites can be divided into two different types: 1. sites that follow the primary amino acid sequence as a continuous or linear interaction site. 2. discontinuous or non-linear binding sites, which are made up of short peptide fragments that are not adjacent in the sequence but are in spatial proximity as a result of folding. Non-linear binding sites predominate in both protein-protein interactions, and in protein binding of small compounds [2]. Their detection is challenging because conventional mapping techniques have limited capabilities [3,4]. The increasing number of structurally-determined proteins often permits a structure-based automated approach to the design of peptide libraries that can mimick particular surface regions. As Atassi et al. [5] and Lee et al [6] proposed, spatially neighbouring sequence segments have to be linked by short (peptidic) linkers to conserve local conformation. To facilitate this process, we have integrated the LIP database containing all peptidic fragments derived from the Brookhaven Protein Data Bank (PDB) up to a length of 15 residues [7]. SUPERFICIAL makes it possible to scan a specific part of the protein or the whole protein. Determination of the peptides and selection of the linkers are automated, and substantial peptide libraries can be generated.

## Implementation

The program was implemented in Delphi and is designed for versions of Windows 98 upwards.

Three problems have to be solved:

1. *Determination *of those parts of the protein surface that provide the basis of the peptide library.

2. *Localisation *of those peptides that are neighboured in space (but not in sequence) and form a potential non-linear binding site.

3. *Detection *of linkers to connect the spatially neighbouring peptides in consideration of the local conformation.

### Determination of the surface segments

At first, the library should contain only peptides that mimic the surface of the protein, or of the selected protein chain. Therefore, the peptides themselves should consist mainly of amino acids that are solvent-accessible. In general, there are several possibilities of defining an amino acid as surface-exposed. One can estimate the proportion of the surface area of an amino acid that is accessible to water [8] and set a threshold for this value. The threshold, however, can be varied for each type of amino acid. Since the packing of protein structures differs depending on the size, degree of polymerisation, and origin of the structure (NMR, crystal or a model), there is no threshold matching all kinds of structures.

**Figure 2:**
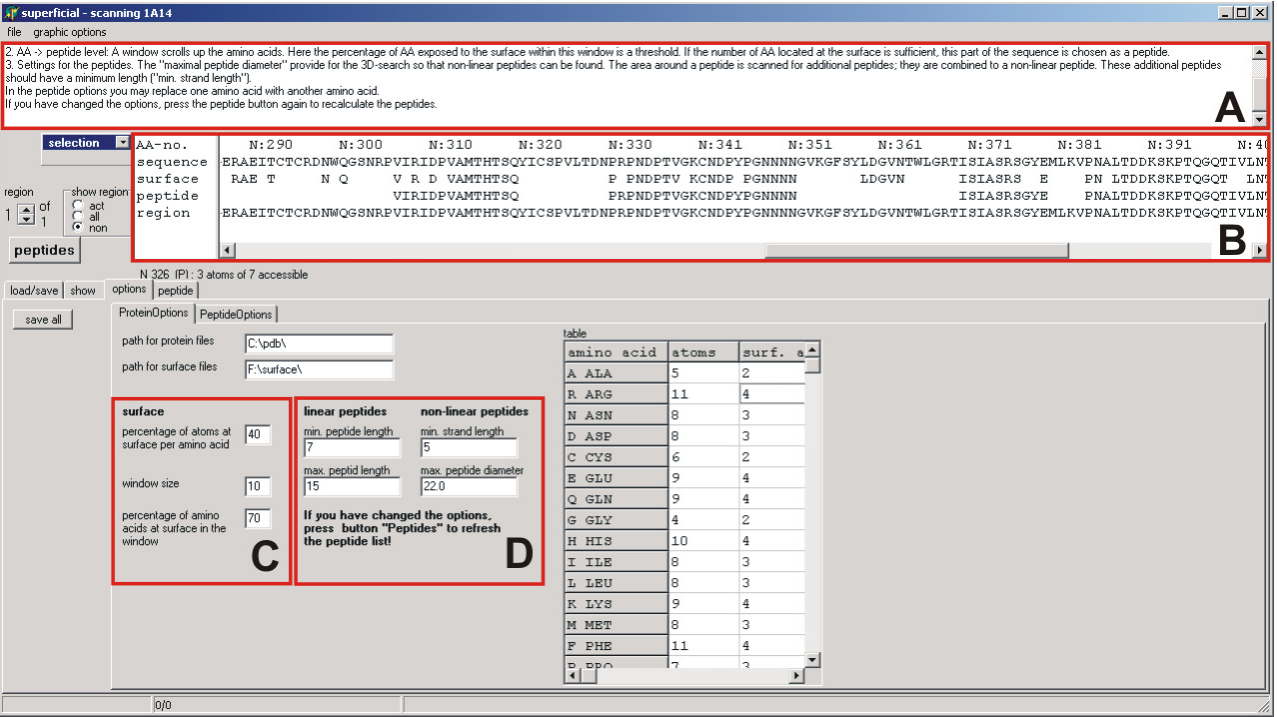
Screenshot of SUPERFICIAL displaying the options. Sections A and B are the same for all submenus/menu items ("load protein", "show", "options" and "peptide"). Section A gives a short description of the options and may act as a guide for the user. In section B the subsequent results of the settings are shown and the user may check the effects on the size of the surface and the peptides. The options in C determine the surface of a protein, whereas the first entry ("percentage of atoms at surface per amino acid") has the greatest influence on the surface extension. Section D gives the definitions for peptide generation. All changes are visualised in B on sequence level. The whole protein is displayed in the submenu "show" (Fig. 3).

SUPERFICIAL meets that challenge by automatically evaluating the solvent-accessibility for each atom. Depending on the proportion of atoms exposed to the surface (Fig. 2, section C and Table), the accessibility of an amino acid is divided into two states – buried (non-accessible) or exposed (accessible). This option can be used to modify the extension of the protein's surface. If only exposed amino acids are considered for the peptide library, the resulting peptides become very small, notably in scanned semi-exposed helical regions; thus small gaps require filling. For this purpose, a sliding-window technique was used. The user defines a window (Fig. 2, section C) that scrolls down the sequence of the surface to close gaps or eliminate detached amino acids. The resulting solvent-accessible sequence segments represent the surface of the protein and therefore provide the basis for the generation of a peptide library. These segments mimic potential linear binding sites, whereas the non-linear binding sites consist of several segments.

### Peptide generation

If only linear peptides are of interest, their length can be defined (Fig. 2, section D). The solvent-accessible sequence segments are then tailored accordingly. The procedure to identify and assemble the non-linear peptides is more sophisticated. Starting from one linear peptide-fragment, the surrounding space is scanned in a user-defined diameter (Fig. 2, section D). Peptide-fragments within this diameter are combined to form a single entity.

#### Search for linkers

To preserve their conformation, the gaps between the peptide-fragments are filled with linkers, short amino acid sequences derived from the LIP (Loops in Proteins) database [7]. The LIP database contains all peptidic fragments from the PDB up to a length of 15 residues. The peptidic fragments obtained from LIP and the peptide-fragments generated by SUPERFICIAL are combined to form the complete non-linear peptides.

The linkers are integrated depending on the distances and angles of the stem atoms, as described in [7]. All possible arrangements of the peptide-fragments of the protein are examined. For each combination the shortest linkers are determined, and the one with the shortest total length is accepted. This procedure may change the order of the peptide-fragments, in case it shortens the linker. Additionally, it minimises the insertion of foreign amino acids.

The current size of the LIP database is approximately 8 Gigabytes, and it contains about 100 million entries. To connect to this database, it is necessary to install this large amount of data. Instead of the whole database, the downloadable version of SUPERFICIAL implements a table that is derived from the LIP database. This table contains a grid of parameters (distances and angles) along with the corresponding number of amino acids necessary to bridge a gap between two peptides. Applying the table instead of the LIP database allows rapid identification of appropriate peptide linkers, though their sequence is arbitrary. Amino acids are represented by the character "X" that can be replaced in praxis by poly-alanine and/or glycine.

## Results and discussion

SUPERFICIAL has been tested on Windows 98, NT, 2000 and XP. Additional visualization tools are not required. It can read files in PDB format, which are either derived from the PDB or from modelling. We have successfully tested proteins up to 50,000 atoms, though the maximum size accepted is dependent on computer memory.

**Figure 3:**
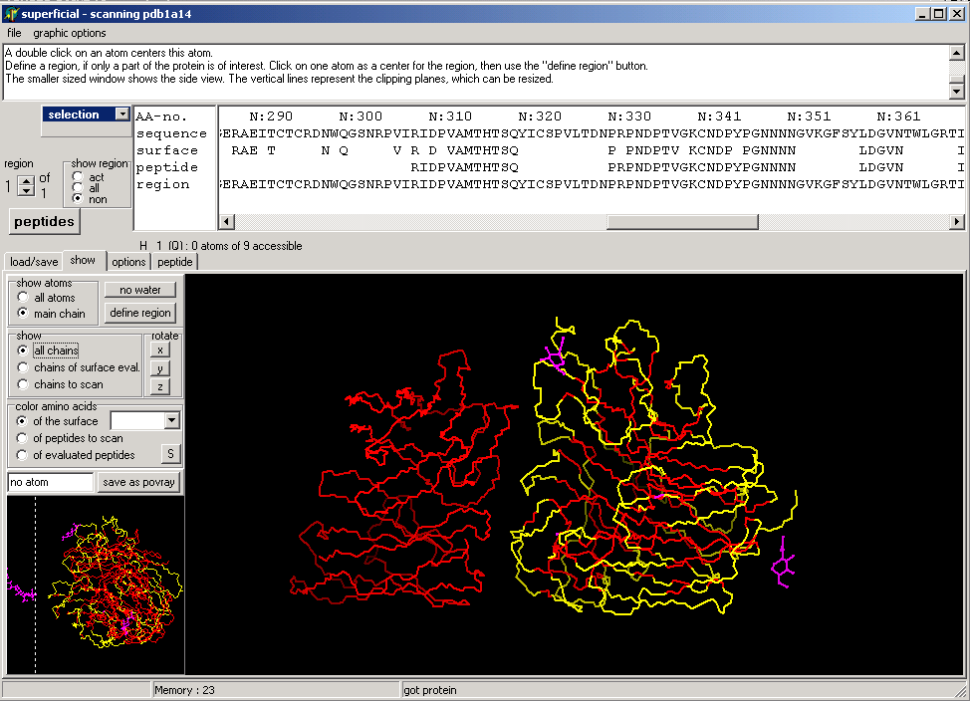
Screenshot of SUPERFICIAL showing the 3D view of the protein. The functionality of this tool is exemplified by the crystal structure of a complex between influenza virus neuraminidase and an antibody (PDB-code: 1a14).

**Figure 4:**
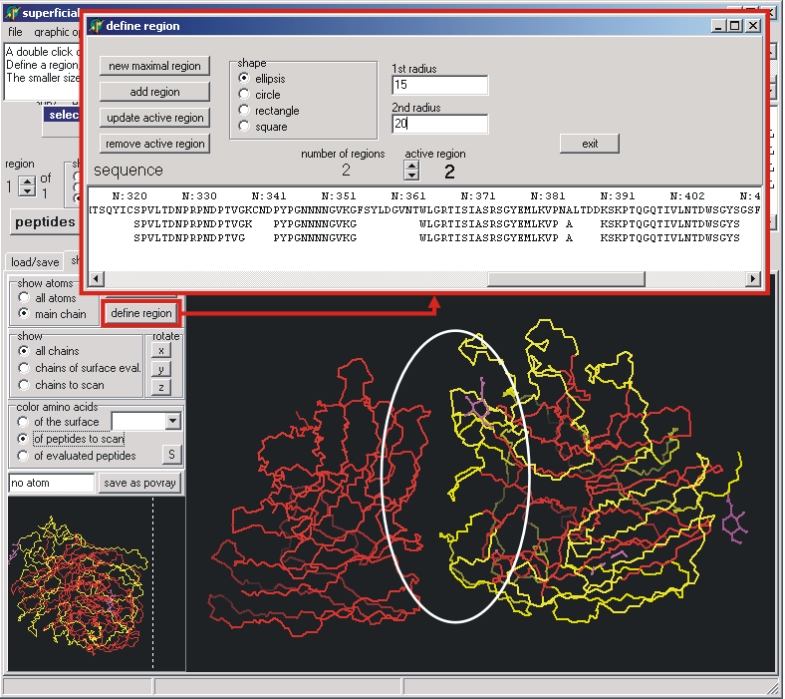
Screenshot to illustrate the selection of a region (white ellipse). The peptide library will be generated for this region only.

SUPERFICIAL automatically defines the protein surface, using preset default values applicable to a range of proteins. To consider the heterogeneity of proteins and for "fine-tuning", the user can choose between various options to specify the surface area (Fig. 2, section A). The user can scan either the entire protein (Fig. 3), selected chains, or a region of specific interest (Fig. 4). The program will only consider the selected part of the protein for scanning and producing a peptide library. All effects of the settings are shown at sequence level in the window above (Fig. 2, section B), and on the annotated 3D structure of the protein (Fig. 3), where the surface is highlighted. When the peptide library is complete, every peptide can be displayed individually and discarded if required. The whole project can be saved and restored at any stage of the process, so different settings can be compared.

To avoid problems during peptide synthesis, amino acids can be automatically replaced, e.g. cysteine versus serine. All generated peptides are listed within a saveable table. Such a structure-based peptide library provides the source for chemically-prepared peptide arrays to identify and characterise binding sites, respectively [9,10].

## General discussion

Atassi et al. [5,11] and Lee et al. [6] proposed the idea of linking several peptides forming a non-linear binding site with short peptidic linkers. They first identified the amino acids of a non-linear antigenic site in native lysozyme and then linked them into a single peptide by inserting glycine residues. A different approach was used by Casset et al. [12], Franke et al. [13] and Eichler [14]. They used circular scaffolds to present the peptides of a non-linear binding site, and these structures maintained the conformation of the peptides found in the original protein. For all these methods, detailed structural information of the binding site or the interacting amino acids has to be available. These problems are overcome with SUPERFICIAL, since only the structure of the protein is required. Determination of the surface and selection of the peptides can be influenced by the user, while the selection of the linker and the generation of the peptide library are automatic. The whole library provides the basis for a high-throughput synthesis (e.g. the SPOT-synthesis [15]) and the identification of binding peptides.

Methods to connect peptides with linkers are mostly used during homology modelling. Generally, two approaches are applied: *ab initio *or knowledge-based methods. *Ab initio *methods usually scan the whole conformational space, while knowledge-based methods search for protein segments with a known three-dimensional structure that fits into a gap. Both methods assess the possible linkers according to potential or scoring functions. For *ab initio *methods, the complexity, and therefore the time and effort increase with the length of the linker. As shown in [7], detection of suitable linkers by means of LIP is usually performed faster and more accurately than by other methods.

Non-peptidic linkers between peptides can also be applied, but in contrast to the 100 million linkers contained in LIP, their number and availability are limited. Therefore, not all possible conformations of peptide-fragments can be conserved with non-peptidic linkers. Currently, there is no public database of non-peptidic structures that can serve as linkers. Although the combination of peptide fragments and non-peptidic linkers or scaffolds can be advantageous if only a small number of structures is to be synthesised, such a method is not applicable for a high-throughput synthesis.

Predictions concerning the nature of antigenicity and binding sites have a large literature. Determining the antigenicity of different proteins implies that such areas share common properties [16]. Mostly, these involve the hydrophilicity, flexibility and accessibility of a protein. The program BEPITOPE, for example, uses such properties to predict linear protein epitopes and rank them according to their hydrophobicity [17]. SUPERFICIAL follows a different approach: the 3D structure of the whole surface, or parts of it, are considered and transformed into a peptide library representing this surface. Currently, it is the only program that identifies potential non-linear binding sites. Even though information on probable binding sites is not given, SUPERFICIAL includes all potential binding sites by examining the entire protein surface.

## Conclusion

SUPERFICIAL is a unique tool for surface mapping, which considers the 3D structure of a protein and translates it into a peptide library. The most novel aspect of this program is its ability to propose peptides that can mimic non-linear binding sites, making it interesting, for instance, in vaccine development.

## Availability and requirements

A free version of SUPERFICIAL is available for academic use at :

• Project name: SUPERFICIAL

• Project home page: 

• Operating system(s): Windows 98 upwards

• Programming language: Delphi

• Other requirements: none

• Restrictions to use by academics: registration needed

• Restrictions to use by non-academics: licence needed

## Authors' contributions

AG created the program, helped to draft the manuscript, web site and demos. ISJ drafted the manuscript, the web site and demos. RP was the coordinator of the project.

**Figure 1:**
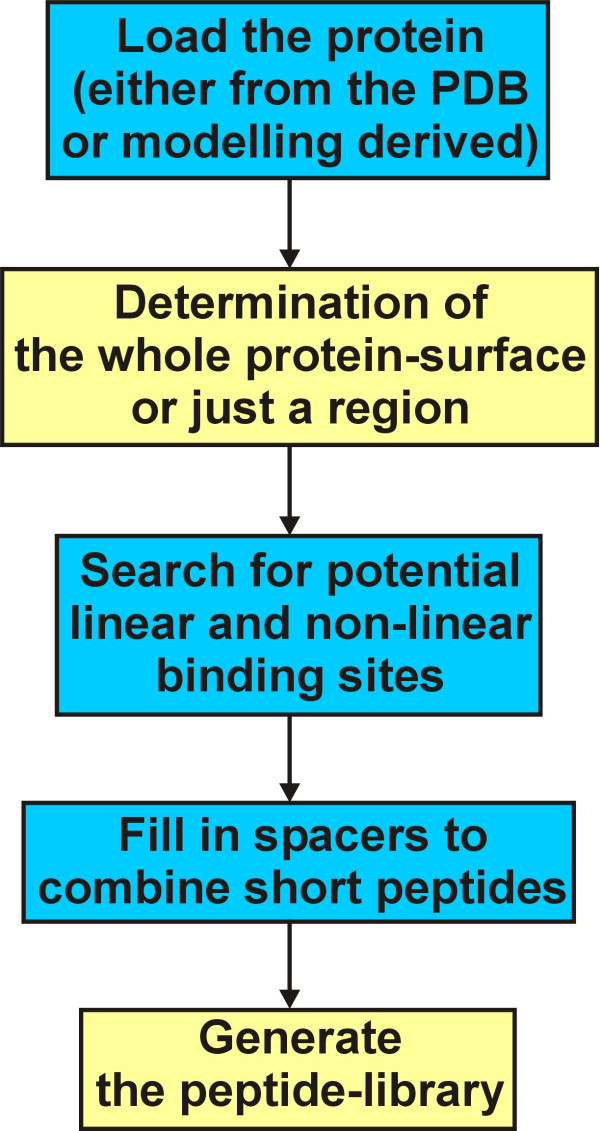
Flow chart to illustrate the process from loading a protein to the generation of the peptide library.
